# Wicking in Porous Polymeric Membranes: Determination of an Effective Capillary Radius to Predict the Flow Behavior in Lateral Flow Assays

**DOI:** 10.3390/membranes12070638

**Published:** 2022-06-21

**Authors:** Patrick Altschuh, Willfried Kunz, Marcel Bremerich, Andreas Reiter, Michael Selzer, Britta Nestler

**Affiliations:** 1 Institute for Digital Materials Science, Karlsruhe University of Applied Sciences, Moltkestr. 30, 76133 Karlsruhe, Germany; andreas.reiter@h-ka.de (A.R.); michael.selzer@kit.edu (M.S.); britta.nestler@kit.edu (B.N.); 2 Institute for Applied Materials–Microstructure Modelling and Simulation, Karlsruhe Institute of Technology, Strasse am Forum 7, 76131 Karlsruhe, Germany; 3 Sartorius Stedim Biotech GmbH, August-Spindler-Strasse 11, 37079 Goettingen, Germany; marcel.bremerich@sartorius.com

**Keywords:** effective capillary radius, wicking, paper-based microfluidics, lateral flow assays, phase-field, COVID-19

## Abstract

The working principle of lateral flow assays, such as the widely used COVID-19 rapid tests, is based on the capillary-driven liquid transport of a sample fluid to a test line using porous polymeric membranes as the conductive medium. In order to predict this wicking process by simplified analytical models, it is essential to determine an effective capillary radius for the highly porous and open-pored membranes. In this work, a parametric study is performed with selected simplified structures, representing the complex microstructure of the membrane. For this, a phase-field approach with a special wetting boundary condition to describe the meniscus formation and the corresponding mean surface curvature for each structure setup is used. As a main result, an analytical correlation between geometric structure parameters and an effective capillary radius, based on a correction factor, are obtained. The resulting correlation is verified by applying image analysis methods on reconstructed computer tomography scans of two different porous polymeric membranes and thus determining the geometric structure parameters. Subsequently, a macroscale flow model that includes the correlated effective pore size and geometrical capillary radius is applied, and the results are compared with wicking experiments. Based on the derived correction function, it is shown that the analytical prediction of the wicking process in highly porous polymeric membranes is possible without the fitting of experimental wicking data. Furthermore, it can be seen that the estimated effective pore radius of the two membranes is 8 to 10 times higher than their geometric mean pore radii.

## 1. Introduction

**Figure 1:**
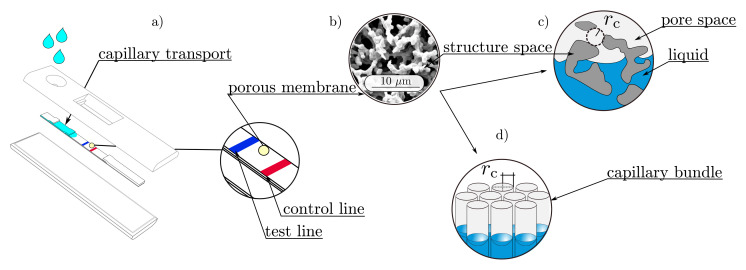
Graphical overview: (**a**) A typical design of a lateral flow assay (LFA), with highlighted test and control lines; (**b**) scanning electron microscopy (SEM) image of a porous polymeric membrane (PPM); (**c**) schematic representation of the pore and the structure spaces [6]; (**d**) Lucas–Washburn assumption of a porous medium, as a bundle of parallel capillaries [6].

Wicking is the surface-driven imbibition process in porous microstructures, in which a non-wetting fluid (gas) is replaced by a wetting one (water), when exposed to a capillary suction pressure. This capillary phenomenon is strongly promoted by an open-pored and porous microstructure, which can be found in many materials, such as textiles, woven fibers, and porous polymeric membranes (PPMs). In particular, PPMs are widely used in lateral flow assays (LFAs), where they function as an autarkic microfluidic pump system that transports a liquid sample, containing analyte and detector particles, toward the detection zone (test and control lines, see Figure 1a).

Due to this quality, cost-effective and easy-to-use LFAs can be realized, covering a wide range of applications in medical diagnostics, drugs of abuse control as well as the environmental monitoring of contamination in water, soil, and air [1]. Particularly in medical diagnostics, they make it possible to stem and control major pandemics, such as the severe acute respiratory syndrome (SARS) outbreak of 2003, which happened in Asia, or the current worldwide COVID-19 outbreak. Thus, by further improving LFAs, an evident contribution to global health is made [2,3].

However, optimizing and designing PPMs for LFAs is challenging for different reasons, such as the fact that (i) multiple length scales are involved, (ii) convection, diffusion, and reaction mechanisms are simultaneously present, and (iii) effective properties of the complex microstructure are difficult to determine. In particular, the prediction of the fluid flow across multiple length scales is crucial, as the flow condition inherently affects the sensitivity of LFAs [4]. In order to bridge the different length scales, the use of appropriate upscaling methods is necessary. For this purpose, bottom-up approaches are particularly suitable [5], whereby the information is passed on from the smallest relevant scale to the next larger scale, by using effective parameters. To enable their application, an accurate determination of the effective parameters is essential. In particular, regarding the wicking behavior in PPMs, the use of simplified analytical flow models requires an accurate effective capillary radius.

Many approaches exist to model the wicking process in porous microstructures. The three most common ones are based on (i) the Lucas–Washburn equation, (ii) Darcy’s law, and (iii) the Richards equation and are extensively discussed in the literature [3,7,8,9,10,11,12]. All approaches require different effective properties of the porous media, such as porosity, permeability, a fiber radius, and a pore radius, while the determination procedure is based on the volume averaging of the properties in a representative volume element (RVE) [13,14]. Particularly for the permeability, this method is widely used by solving the Stokes equation in the pore space. Thus, numerous correlations between the porosity, the specific surface area, and a resulting permeability have been reported [15,16,17,18].

However, little attention has been paid to the averaged pore radius in complex porous microstructures, although it is important for the description of the capillary suction pressure. As a driving force, this pressure is usually modeled using the Young–Laplace equation, in which the pressure difference acting across a curved free surface or interface between two immiscible fluids is expressed as

(1) $$\Delta P_{\mathrm{cap}}=\frac{2\gamma_{\mathrm{lg}}\cos\left(\theta_{e}\right)}{r_{c}}.$$

Here, $\gamma_{\mathrm{lg}}$ describes the surface tension between the acting fluids and $\theta_{e}$ is the equilibrium contact angle. The main assumption behind Equation (1) is that the capillary pressure is developed in a cylindrical capillary with the radius $r_{c}$. Lucas and Washburn transferred this assumption to a porous medium, by assuming it to be a bundle of aligned capillaries (see Figure 1d)), each having the same capillary radius [19]. According to this assumption, the curved free surface in a cylindrical capillary with a mean surface curvature *H* can be correlated with the capillary radius $r_{c}$, by

(2) $$\begin{array}{c} H=\frac{\cos\left(\theta_{e}\right)}{r_{c}} \end{array},$$

where the capillary radius $r_{c}$ can also be considered as the geometric pore radius (see Figure 1c). As long as the perimeter of the cross section in the capillary is closed and axisymmetric, the Young–Laplace equation can be applied and analytical expressions are available [20,21]. However, the cross sections of the complex structures are not always closed, and it is unlikely that they are axisymmetric. Furthermore, flow paths are not aligned in parallel tubes but show tortuous curves. Therefore, these assumptions are generally not satisfied for the Young–Laplace equation for PPMs. Thus, a distinction must be made between the geometric and the effective pore radius [19] of complex porous structures.

Instead of accurately determining the effective pore radius, it is common to fit the mathematical wicking models to the experimental results and derive the effective pore radius or the capillary suction pressure [19,22,23,24,25,26]. The resulting effective pore radius can be used to accurately describe the fluid flow for one specific PPM; however, prediction attempts for new wicking structures always require further experiments.

Geometry-based approaches to determine an effective pore radius require a detailed representation of the pore space, which can be obtained by scanning electron microscopy (SEM), transmission electron microscopy (TEM), confocal laser scanning microscopy, or X-ray computer tomography (CT) measurements [27], among others. Alternatively, digital representations can be approached by algorithmically generating both simplified and complex capillary pore spaces [28,29]. For the extraction of the effective pore radius, image processing methods are used to capture morphological properties by voxel-based algorithms. Fitting spheres in the pore space is one approach where the geometric mean radius [30,31] of the spheres is considered as the effective pore radius (see Figure 1c).

Another approach focuses more on structural space and assumes that the porous wicks consist of spherical particles. Since the particle sizes vary along the ligaments, an effective pore radius is derived on the basis of the particle size distributions [9]. As a third approach, it is common to determine the pore space volume and the surface of the structural space, to thus calculate the hydraulic pore radius as an effective radius [32,33,34]. In [12,32], a comprehensive summary of different approaches to estimate an effective pore radius for the wicking process is given.

Approaches based on physical two-phase simulations are reported in the literature as well. Both closed capillaries with different cross-sectional shapes [29] and open-pored porous structures [35,36,37] were generated, while the resulting mean surface curvature of the fluid and gas interface was studied. As a main result of the investigations, it was shown that the resulting mean surface curvature is influenced by the cross section and thus affects the effective pore radius [29].

In this work, two highly porous nitrocellulose membranes (porosities ≈ 85%) are investigated as polymeric wicks, where each shows a complex microstructure and arbitrary cross sections with open perimeters of the pores. The objective of this work is to use physical two-phase simulations to establish a correlation between geometric properties and an effective pore radius, so as to predict the capillary driven wicking process. For the derivation, high-resolution X-ray computer tomography experiments were conducted, and 3D representations of the two membranes were obtained. In our previous work [27], a characterization tool was presented, which allows extracting both the pore and the ligament radius distributions of porous microstructures as the basic geometric properties.

Based on a free energy minimization approach [38,39,40], mean surface curvatures in simplified porous 3D geometries with defined geometric properties are calculated and correlated with an effective pore radius. A final validation of the correlation is done by applying an analytical model with the derived effective pore radii and by comparing the prediction to wicking experiments. All applied simulation methods and characterization tools are implemented in the simulation framework Pace3D [41]. The presented determination of an effective capillary radius is based on the PhD Thesis [6], which is written in German. In order to extend the reach to a broader audience, some results of the work are presented in this article.

## 2. Materials and Methods

### 2.1. Mathematical Modeling

#### 2.1.1. Two-Phase Phase-Field Approach

Wetting phenomena in porous media can be explained from (i) a mechanical and (ii) an energetic point of view. From a mechanical point of view, the pressure drop across a curved surface (suction pressure) is considered as the driving force, and the fluid is sucked into the pore space. In terms of free energies, spontaneous wetting arises from the difference in the total surface energies $\Delta \gamma =\gamma_{\mathrm{sg}}-\gamma_{\mathrm{sl}}$, where $\gamma_{\mathrm{sg}}$ and $\gamma_{\mathrm{sl}}$ represent the interfaces of a solid substrate (s) and the fluids involved represent the gas (g) and the liquid (l), respectively. Thus, with a positive difference in the surface energies Δγ>0, the total free energy can only be minimized by wetting the substrate with the liquid. Both views are linked by Young’s familiar law $\gamma_{\mathrm{lg}}\cos\left(\theta_{e}\right)=\gamma_{\mathrm{sg}}-\gamma_{\mathrm{sl}}$, which gives the ratio of the surface energies as the cosine of the equilibrium contact angle $\theta_{e}$ [42].

The wetting process as well as the evolution of free surface curvatures in open-pored cross sections can be investigated in detail by applying a two-phase phase-field approach, which is based on a Ginzburg–Landau free energy density functional [38,40]. In this approach, the two order parameters $\phi_{g}\left(x,t\right)$ and $\phi_{l}\left(x,t\right)$ are introduced for each of the two phases (gas and liquid), with their values varying from 1 inside to 0 outside the bulk of the respective phase. Since the order parameters fulfill the side condition of $\phi_{l}\left(x,t\right)+\phi_{g}\left(x,t\right)=1$, only one order parameter $\phi \left(x,t\right)=\phi_{l}\left(x,t\right)=1-\phi_{g}\left(x,t\right)$ is sufficient to describe the investigated two-phase system.

In the considered phase-field approach (e.g., [39]) a diffuse interface is formed between the two phases. Here, the order parameter continuously varies from ϕ(x,t)=1 (in the liquid) to ϕ(x,t)=0 (in the gas), allowing the position of the interface to be tracked in space and time. Additionally, the model is extended by a wetting boundary condition [39], which accounts for the difference in the surface energies Δγ on the substrate and enables the description of wetting phenomena. The applied two-phase phase-field approach reads as

(3) $$F\left(\phi \right)=\int_{\Omega }\left(\epsilon \gamma_{\mathrm{lg}}{\left|\nabla \phi \right|}^{2}+\frac{1}{\epsilon }w\left(\phi \right)+f_{g}\left(\phi \right)\right)d\Omega +\int_{\partial_{s}\Omega }f_{w}\left(\phi \right)\;dS.$$

Here, Ω is the spatial domain and ϵ is a parameter related to the thickness of the diffuse interface. The gradient energy density $\gamma_{\mathrm{lg}}{\left|\nabla \phi \right|}^{2}$ and the multi-obstacle potential w(ϕ) together reflect the free surface energy of the liquid–gas interface, while $f_{g}\left(\phi \right)=g\;\cdot \;x\rho I\left(\phi \right)$ represents the hydrostatic pressure depending on the interface position in space x and the acting gravitational body force g. The energy formulation $f_{w}\left(\phi \right)$ is used to model the energy contributions on the substrate surface $\partial_{s}\Omega$ as follows:

(4) $$f_{w}\left(\phi \right)=\gamma_{\mathrm{gs}}+\left(\gamma_{\mathrm{ls}}-\gamma_{\mathrm{gs}}\right)I\left(\phi \right).$$

Here, the function $I\left(\phi \right)=\phi^{3}\left(6\phi^{2}-15/\phi +10\right)$ interpolates the values across the diffuse interface [39]. A simple analysis across the liquid–gas interface and along the substrate surface shows that the applied wetting boundary condition fulfills Young’s law [39]. The time- and space-dependent evolution of the interface results from the minimization of the free energy functional in Equation (3), using variational calculus methods. The resulting partial differential equations are known as the Allen-Cahn equations and can be written as follows:

(5) $$\tau \epsilon \frac{\partial \phi }{\partial t}=2\epsilon \gamma_{\mathrm{lg}}\Delta \phi -\frac{1}{\epsilon }\frac{\partial w}{\partial \phi }\left(\phi \right)-\frac{\partial f_{g}}{\partial \phi }\left(\phi \right),\;\;\mathrm{in}\;\;\Omega ,$$

where the wetting boundary condition reads as

(6) $$-2\epsilon \gamma_{\mathrm{lg}}\nabla \phi \;\cdot \;n+\left(\gamma_{\mathrm{gs}}-\gamma_{\mathrm{ls}}\right)\frac{\partial I}{\partial \phi }=0\;\;\mathrm{on}\;\;\partial_{s}\Omega .$$

Here, the normal vector to the substrate surface $\partial_{s}\Omega$ is denoted as n.

#### 2.1.2. Macroscopic Flow Model for Wicking Processes

The main motivation for using macroscopic flow models is to supply simple analytical equations for a rather complex flow problem. As a second important point, macroscopic flow models allow bridging different length scales.

Three main approaches are used to model the flow, which are all based on the description of the transport process by including the dominant effects, such as friction, gravity, and capillarity in the momentum balance equation. Since porous membranes are assumed to be fully wetted, and their complex structure cannot be approached by a bundle of aligned capillaries, a model based on Darcy’s law is applied [7]. Here, the force balance is expressed as follows:

(7) $$\rho \frac{d(h\dot{h})}{dt}+\frac{\varphi }{K}\eta h\dot{h}+\rho gh=\frac{2\gamma_{\mathrm{lg}}\cos\left(\theta_{e}\right)}{r_{\mathrm{eff}}}.$$

Inertial, viscous, and gravitational forces (the first, second, and third terms in Equation (7)) balance the capillary force (fourth term in Equation (7)), where the dynamic viscosity η, the surface tension $\gamma_{\mathrm{lg}}$, and the contact angle $\theta_{e}$, respectively, represent the properties of the fluid and the fluid/substrate interaction. The effective parameters of the microstructure are expressed with the permeability *K*, the porosity φ, and an effective pore radius $r_{\mathrm{eff}}$. The effective pore radius is the focus of this work. The propagation distance *h* and the propagation velocity $\dot{h}$ of the fluid are compared with experimental results as characteristic parameters. Depending on the ratio of the involved forces, some terms in Equation (7) can be neglected. A common way of characterizing the dominant forces is by estimating the Bond number [43]

(8) $$Bo=\frac{\mathrm{Body}\;\mathrm{force}}{\mathrm{Surface}\;\mathrm{tension}\;\mathrm{force}}=\frac{\rho gh_{\max}L}{2\gamma_{\mathrm{lg}}}$$

and the Weber number [44] with

(9) $$We=\frac{\mathrm{Inertial}\;\mathrm{force}}{\mathrm{Surface}\;\mathrm{tension}\;\mathrm{force}}=\frac{\rho {\dot{h}}^{2}L}{\gamma_{\mathrm{lg}}},$$

where *L* is the characteristic length scale of the system and $h_{\max}$ describes the maximum vertical height of a rising water column. In the case of the PPMs of interest, the geometric pore size (from 1μm–10μm) is taken as the characteristic length scale *L*. Moreover, in the wicking experiment performed in this study (see Section 2.3), the maximum vertical height of a membrane sample is $h_{\max}=4\mathrm{cm}$ and the liquid used has a relatively high density and low surface energy, compared to water (see Table 1). These assumptions lead to a low Weber number that is much smaller than 1 (We≪1) but result in a Bond number of Bo =0.22, which is not clear evidence for the dominance of surface tension forces.

This means that inertial forces can be neglected, while gravitational body forces should be included. The resulting expression is a force balance between viscous, gravitational, and capillary forces:

(10) $$\frac{\varphi }{K}\eta h\dot{h}+\rho gh=\frac{2\gamma_{\mathrm{lg}}\cos\left(\theta_{e}\right)}{r_{\mathrm{eff}}}.$$

For the applied force balance, a fully analytical solution is given in [7] as follows:

(11) $$h\left(t\right)=\frac{1}{c}\left[1+W(-e^{-1-\frac{c^{2}t}{b}})\right].$$

Here, W(x) is the Lambert W function and the variables *b* and *c* represent two coefficients for the gravity and viscosity terms, respectively, [8]. They can be defined as

(12) $$b=\frac{r_{\mathrm{eff}}}{2\gamma_{\mathrm{lg}}\cos\left(\theta_{e}\right)}\frac{\varphi \eta }{K}$$

and

(13) $$c=\frac{r_{\mathrm{eff}}}{2\gamma_{\mathrm{lg}}\cos\left(\theta_{e}\right)}\rho g.$$

Equations (11)–(13) are used as a macroscopic model to predict wicking, while the effective properties are extracted from 3D digital twins of the porous polymeric membranes.

### 2.2. Digital Twins of Porous Polymeric Membranes

The investigated PPM is a commercially available, unsupported and impregnated nitrocellulose membrane for lateral flow assays (UniSart^®^ CN140, Sartorius Stedim Biotech GmbH, Göttingen, Germany). As specified by the manufacturer, it has a nominal pore size of 8 μm and a thickness of ∼135 μm, while the impregnation of the intrinsic surface provokes the hydrophilic wetting behavior. For this work, two different lots (sample 1 and sample 2) of the membrane are used for both the simulation-based and the experimental analysis. For the pore-scale simulations, 3D data of the microstructures are extracted from CT measurements.

#### 2.2.1. High-Resolution Computer Tomography (Nano CT)

**Figure 2:**
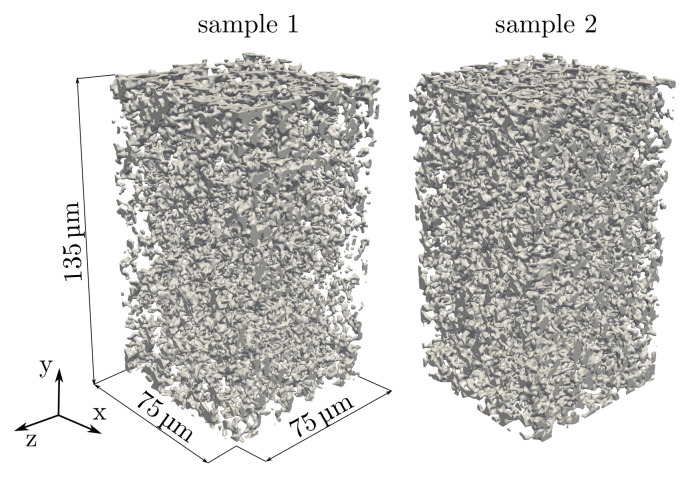
Reconstructed porous microstructures of the two different PPM test samples (sample 1 and sample 2), in which the gray parts show the solid membrane. Each of the presented samples exhibits a volume of (75×135×75)μm. Sample 1 corresponds to the SEM image in Figure 1.

3D data of the PPMs are obtained by high-resolution X-ray computer tomography (nano CT) measurements. For the experiments, the beamline ID16B-NA at the European Synchrotron Radiation Facility (ESRF) in Grenoble has been used [45]. The reconstructed 3D representation of the data consists of 900 equally spaced 2D images with a spatial resolution of 150 nm.pixel-1. The stack of 8-bit (256 intensity levels) grayscale images is filtered by a Gaussian 3D filter with a sigma of three voxels. Following this, a binarization algorithm is applied, in which the threshold is adjusted to obtain the experimentally measured porosity as described in [27]. For both processing steps, the image processing software ImageJ v1.51J8 [46] is used. As a result, two digital twins of the porous microstructures with (500×900×500) voxels are obtained, giving (75×135×75)μm in physical units and reflecting the total membrane thickness with ∼135 μm. The digital representations for sample 1 and sample 2 are shown in Figure 2. As the SEM image in Figure 1b) and the nano-CT scans show, the complex microstructures do not have circular and closed pores.

#### 2.2.2. Effective Properties

The four main effective properties for describing the fluid flow in porous wicks are the porosity φ, the mean pore radius $r_{c}$, the mean ligament radius $r_{l}$, and the permeability *K*. While the porosity is determined experimentally, all other properties are extracted from the introduced 3D microstructures on the pore scale, by using the simulation framework Pace3D [41]. In order to extract representative effective properties, the entire section of 500×900×500 voxels was used to determine the parameters for each sample.

The experimental measurements of the porosities were performed by weighing the dry and water-wetted membranes as described in [27]. These porosities are used to adjust the filter algorithms accordingly. Subsequently, the obtained porosities in the reconstructed 3D structure are verified by calculating the volume of the pore space and dividing the result by the total volume. Both are measured on the basis of voxels.

**Figure 3:**
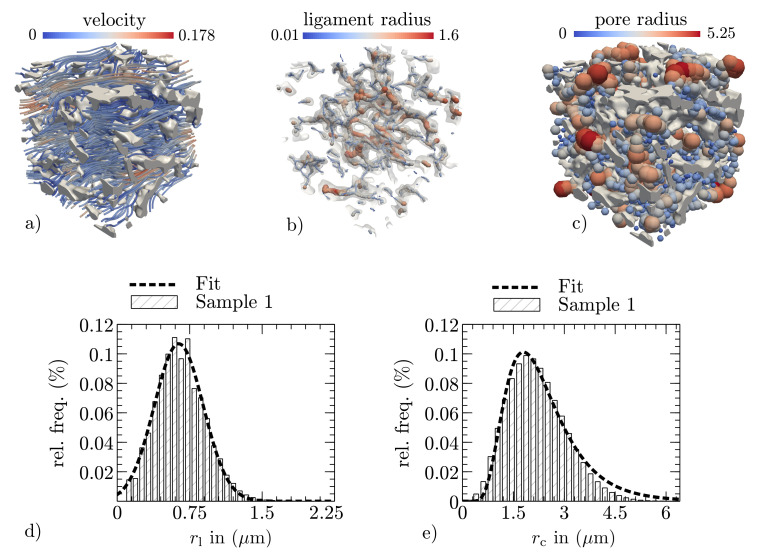
Exemplary extraction of effective properties in a 30×30×30μm section of membrane sample 1: (**a**) the resulting velocity distribution of the fluid flow simulation; (**b**,**c**) visualization of the local ligament and pore radius detection, realized by combining a Euclidean distance map and a thinning algorithm as described in [27]; and (**d**,**e**) the resulting ligament and pore radius distributions for sample 1 (shown for the complete reconstructed sample).

As a measurement of the conductivity of Newtonian fluids, the permeability describes the resistance of porous materials to fluid flow forces. In general, the permeability K is a symmetric tensor of second rank [47]. Since the wicking possess a main flow direction, which is for the following description assumed to be in the x-direction, only mean values are considered, while corresponding indices in the equation are neglected. To determine the permeability, fluid flow simulations are performed in the pore space by defining a pressure difference Δp across the considered PPM layer with a thickness *s* and solving the Stokes equations for the steady-state velocity distribution $v={\left(u_{x},\;u_{y},\;u_{z}\right)}^{T}$, as shown in Figure 3a. By applying Darcy’s law, the permeability is then calculated as follows:

(14) $$K=\frac{\eta Us}{\Delta p},$$

where η describes the dynamic viscosity and *U* denotes the Darcy velocity in the main flow direction (e.g., $U=\varphi {\bar{u}}_{x}$).

The geometric mean pore radius $r_{c}$ and the mean ligament radius $r_{l}$ of the porous membrane are referred to as structure parameters. They are estimated by an image analyzing method developed and implemented in Pace3D and applied in both the pore and the structural space of the 3D microstructure. Due to the combination of a Euclidean distance map and a thinning algorithm, the method is able to estimate the local distributions of the pore sizes and the local ligament radius (see Figure 3b,c). Based on the local distributions shown for the ligaments and the pore sizes in Figure 3d,e), the mean values for the structure parameters are calculated.

The methods for obtaining the effective properties are described in more detail in [27]. The determined effective properties for both samples are summarized in Table 1.

### 2.3. Wicking Experiment

**Figure 4:**
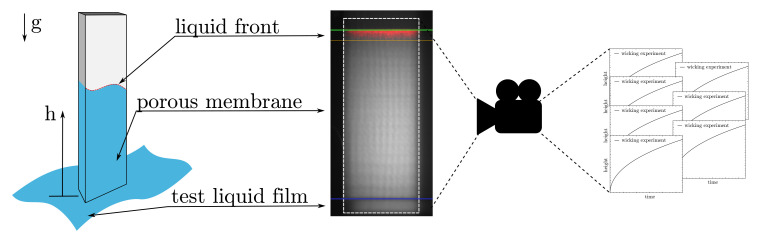
Schematic description of the wicking experiment (**left**) and a real image of the detection of the liquid front, taking place during the experiment (**right**).

Wicking experiments are commonly used to check the quality during the industrial production process and to divide the membranes into different wicking speed categories. A standardized experiment setup was used to determine the wicking behavior. For this purpose, membrane samples with a size of 25 × 75 mm are clamped in a suspension device. With their shorter edge, the samples are then inserted into a thin film of the test liquid, and the porous microstructures are wetted by capillary forces (see Figure 4 (left)). The height *h* of the propagating liquid front is detected by imaging (Figure 4 (right)) and plotted over time *t*.

The time it takes to wet the distance of 40 mm is the so-called wicking time $t_{w}$, while the course of the curve reveals the characteristic wicking behavior. *Porefil^®^* is used as the test liquid, which is commonly applied as the wetting fluid for capillary flow porometry measurements [48,49]. It belongs to the perfluoroethers that have a low surface tension and a contact angle of zero, thereby, resulting in a slow wicking behavior, which is advantageous for the detection of the liquid front. As a major advantage of *Porefil^®^*, it can be assumed that the impregnation does not have a strong influence on the wicking behavior, which would occur during wicking processes with water [50]. Thus, the dynamic influence on the contact angle or the surface tension can be neglected.

Furthermore, it is assumed that, when using *Porefil^®^* during the wicking experiment, neither evaporation nor swelling effects occur, and only the structural and fluid properties influence the wicking effect. Consequently, *Porefil^®^* provides controlled conditions that allow the validation of the effective pore radius. In Table 1, the most important properties of the liquid are listed.

## 3. Results

### 3.1. Simulation of Surface Curvature Formation in Two-Phase Equilibrium Conditions

The validation of the model for two-phase equilibrium conditions is achieved by simulating the rise of a liquid column in capillaries with different cross-sectional shapes. The analytical equilibrium heights are obtained by following Jurin’s law [20]:

(15) $$h_{e}=\frac{2\gamma_{\mathrm{lg}}\cos\left(\theta_{e}\right)}{\rho_{l}gr_{c}},$$

where the capillary driving force is balanced with the gravitational force. Here, $\rho_{l}$ is the density of the liquid and *g* represents the gravitational acceleration.

**Figure 5:**
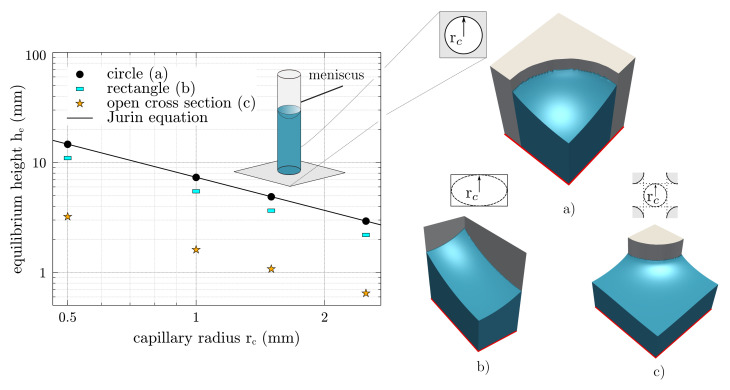
Equilibrium height *h*${}_{e}$, over capillary radius *r*${}_{c}$, for three different cross-sectional shapes in log–log scale: (**a**) circle, (**b**) rectangle, and (**c**) open cross section (adapted from [6]). The simulation results are presented by symbols, while the solid line follows Equation (15) for the circular cross section. The capillary radius $r_{c}$ is defined as half of a minimum measurable distance between two boundaries within the cross section. The red line defines the zero level and the Neumann boundary condition.

For the numerical experiments, water with a density of $\rho_{l}=1000\mathrm{kg}m^{-3}$ is assumed to make the capillary rise, and a free surface is shared with the air, where the interface has a surface tension of $\gamma_{\mathrm{lg}}=72\mathrm{mN}m^{-1}$. According to the conditions on the ground, the gravitational acceleration corresponds to $g=9.81ms^{-2}$. Furthermore, different capillaries with a circular, a rectangular, and an open-pored cross section are shown in Figure 5a–c.

The capillary radius $r_{c}$ is defined as half of a measurable minimum distance between two boundaries within the cross section. Finally, the size of the capillary radius $r_{c}$ is varied in the range of 0.25 mm to 1.5 mm, while the equilibrium contact angle is kept constant at $\theta_{e}=60{}^{\circ }$.

Since we are interested in the equilibrium height, the numerical experiments are conducted by solving only the phase-field equations numerically (Equations (5) and (6)), without any coupling to fluid flow models, such as the Navier–Stokes equations. For this purpose, an explicit Euler scheme is solved for the temporal derivatives, and the finite difference method on an equidistant mesh is used for the spatial derivatives. As the capillaries are assumed to be symmetrical, the simulation domain can be reduced to a quarter of the cross section (see Figure 5a).

A Neumann boundary condition with the normal derivatives of the order parameter ∂ϕ/∂n=0 is defined on the symmetry planes. For the differently shaped capillary cross sections, Figure 5 shows the resulting equilibrium heights *h* over the capillary radius $r_{c}$ in log–log scale. The symbols indicate the simulations, whereas the solid lines correspond to the analytical prediction. According to Equation (15), *h* decreases with increasing $r_{c}$ as also shown by the simulations.

By drifting away from the ideal circular cross section, the equilibrium height changes, even though the geometric capillary radius is equivalent. For the open cross section, the equilibrium height is the lowest, which is due to the deviation of the curvature of the free surface.

First, it should be mentioned that these numerical experiments confirm that the applied phase-field approach is able to predict equilibrium conditions for capillary wetting. Second, the numerical experiments emphasize the importance of taking surface curvatures into account, to thus determine the effective pore radii.

### 3.2. Methodological Determination of the Effective Pore Radius

In order to determine the effective pore radius $r_{\mathrm{eff}}$ for the highly porous membranes, a correction factor *F* is introduced. This factor accounts for the deviation of the surface curvature within open-pored porous structures, compared to the surface curvature in a cylindrical capillary. It is derived by performing a simulation-based parameter study in simplified geometries, for which the presented phase-field approach is applied.

**Figure 6:**
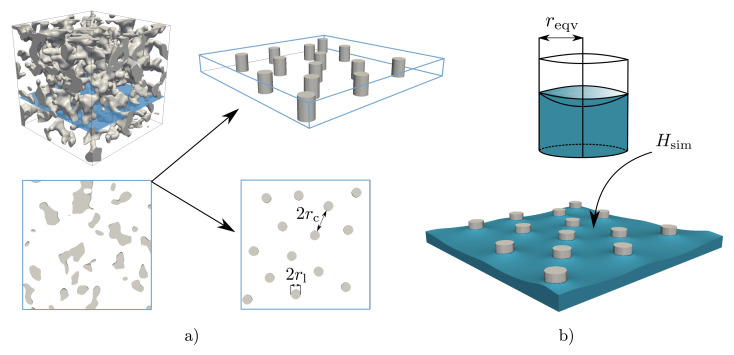
(**a**) Section of a complex membrane of the computer tomography data and the simplified model for the derivation of a correction function. (**b**) Schematic representation of the derivation of the equivalent radius $r_{\mathrm{eqv}}$, based on the simulated mean curvature $H_{\mathrm{sim}}$ (adapted from [6]).

As shown in Figure 2, the investigated polymer membranes reveal a highly porous pore system. Since the capillary pressure is the driving force for the wetting dynamics and is governed by the surface curvature, the correction function *F* is derived by correlating the geometric properties with the resulting surface curvature. Therefore, a simplified open-pored pore system was generated by means of substitute parameters, which is formed from parallel ligament structures oriented in the direction of wetting. The ligament radius $r_{l}$ and the distance between the ligaments $r_{c}$ are introduced as substitute parameters for the mean ligament radius and the mean pore radius. Figure 6a shows a small section (30×30×30)μm of the complex membrane structure as an example and links this to the simplified model with the defined equivalent geometry parameters.

Based on the substitute parameters, a simulation study was conducted, in which a wetting scenario was simulated for each geometry. For this purpose, a total of four simplified geometries with a domain size of (500×500×50)cells were generated, whereby the distance of $r_{c}$ = 80 cells was kept constant and the diameter of the ligaments $r_{l}$ varied between 10 cells and 40 cells. Thus, the ratio of the geometry parameter $r_{l}/r_{c}$ varies between 0.125 and 0.5, which covers the real structural conditions of the CT data, as presented in Table 2.

For each combination of the structure parameter, a two-phase simulation based on Equation (5) was performed. By defining a wetting (water) and a dewetting (air) phase with an initial saturation of 0.5, a meniscus develops between the phases, when equilibrium conditions are reached. Since the capillary length $\lambda_{c}={\left(\gamma_{\mathrm{lg}}/\rho g\right)}^{0.5}$ for *Porefil^®^* is 0.95 mm and therefore consequently larger than the mean pore radii for diagnostic membranes (1μm to 10μm), it is assumed that gravity has no influence on the shape of the equilibrium meniscus.

Therefore, the gravitational term in Equation (5) is neglected for the parameter study and a term that ensures the volume preservation of each phase is used instead, as described in [39]. Furthermore, a periodicity of the investigated geometries is assumed. Since the effective pore radius is a geometric measure, it is assumed to be independent of the contact angle. Therefore, and for validation reasons (see Section 2.1.1), the equilibrium contact angle $\theta_{e}=60{}^{\circ }$ is defined. After reaching the state of equilibrium, the mean curvature of the surface $H_{\mathrm{sim}}$ is determined numerically.

Using the predefined $\theta_{e}$ and the estimated mean surface curvature $H_{\mathrm{sim}}$ for each combination of the structure parameter, an equivalent capillary radius $r_{\mathrm{eqv}}$ is estimated as follows:

(16) $$r_{\mathrm{eqv}}=\frac{\cos\left(\theta_{e}\right)}{H_{\mathrm{sim}}}.$$

This equivalent radius $r_{\mathrm{eqv}}$ can be considered as the radius of a corresponding capillary with a circular cross section and a closed perimeter, as seen in Figure 6b. Hence, $r_{\mathrm{eqv}}$ fulfills the Young–Laplace assumptions. In order to obtain a general correction factor *F*, the equivalent radius is divided by the measured minimum distance between the ligaments $r_{c}$

(17) $$F=\frac{r_{\mathrm{eqv}}}{r_{c}}.$$

**Figure 7:**
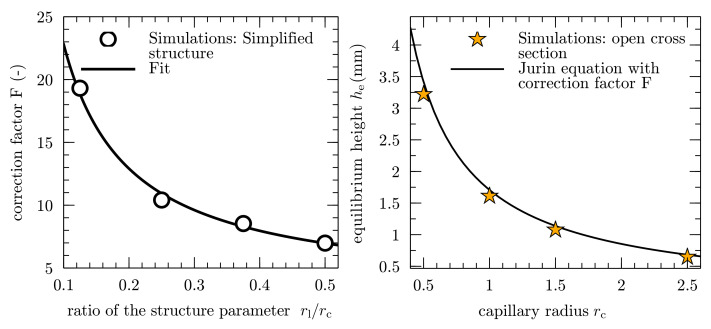
Derivation of an analytical correlation of the correction factor *F*, based on the simulation results (left, adapted from [6]) and equilibrium height $h_{e}$, over ligament radius $r_{c}$, for the open cross-sectional shape of Figure 6b. The right diagram shows the Jurin equation combined with the correction factor F and the simulations from Figure 5c. The simulation results are represented by star symbols, and the solid line follows Equation (20).

As a result, this correction factor can be directly linked to the ratio of the two structure parameters, as shown in the left diagram in Figure 7. For an analytical correlation, the results from the simulations of the simplified geometries are fitted to the following expression:

(18) $$F\left(r_{l},r_{c}\right)=\frac{a}{r_{l}/r_{c}}+b,$$

by using a nonlinear least-squares Marquardt–Levenberg algorithm, which is implemented in the scientific graphing utility Gnuplot 5.2 [51]. The respective best-fit coefficients for the simplified geometries are a=1.980 and b=3.012. The left diagram in Figure 7 shows an inversely proportional behavior of the correction factor *F*, with an increasing ratio $r_{l}/r_{c}$ of the structure parameters. By decreasing the ratio, the correction factor strongly increases, which is caused by a progressing deviation between the equivalent capillary radius $r_{\mathrm{eqv}}$ and the measurable distance between the ligaments $r_{c}$.

The correction factor for the two investigated membranes is estimated by applying the structure parameter from Table 1 and Equation (18). The resulting factors for sample 1 and sample 2 are 9.60 and 7.75, respectively. By multiplying the factors with the measured mean pore radius $r_{c}$ of the porous structure (as described in Section 2.2.2)

(19) $$r_{\mathrm{eff}}=F\left(r_{l},r_{c}\right)r_{c},$$

the effective pore radii of 20.7 μm and 13.4 μm are obtained. An experimentally determined correction factor of ∼7, which lies between the geometric and effective pore radius for the porous structure under investigation, is also reported in the literature [24].

### 3.3. Validation in an Ordered and Open Cross Section

As a first proof of concept, the equilibrium height $h_{e}$ for the open cross section in Figure 5c is calculated by including the correction factor *F* in Equation (15) as follows:

(20) $$h_{e}=\frac{2\gamma_{\mathrm{lg}}\cos\left(\theta_{e}\right)}{\rho_{l}gr_{c}F}.$$

In this case, the ligaments are arranged in an ordered manner and the ratio of the structure parameter $r_{l}/r_{c}=1$ is constant. This results in a correction factor of F=4.3, which means that the equilibrium height for the open cross section is lowered by a factor of 4.3, compared to the circular cross section.

For different minimum ligament distances $r_{c}$, Figure 7 (right) shows the comparison between the simulation results and Equation (20). The simulations and the prediction with Equation (20) show a good agreement, with a maximum deviation of 6.01.

### 3.4. Prediction of Wicking in the PPMs

**Figure 8:**
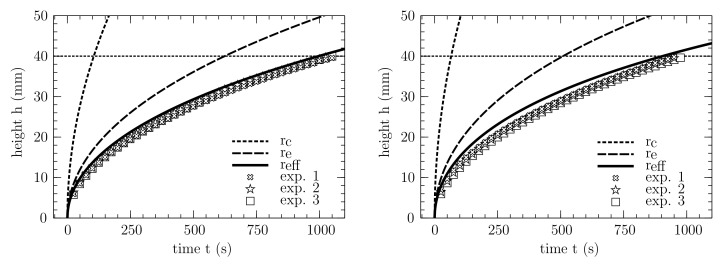
Analytical wicking prediction of three different approaches shown with dashed and solid line and wicking experiments for sample 1 (**right**) and sample 2 (**left**). The typical wicking behavior is presented in a plot in which the height *h* is plotted over the time *t*. The experiments were conducted with *Porefil^®^*. The analytical expression is taken from Equation (11), while the properties are taken from Table 1 and Table 2.

For the two investigated porous membrane samples, the measured wicking times $t_{w}$ are depicted in Figure 8. The experiments for both samples are performed with *Porefil^®^*. For each sample, three wicking curves were determined. Since the corresponding measurement curves lie on top of each other and there is a small relative standard deviation for both samples, it is clear that the measurements are reproducible. Therefore, the results are used to validate the presented approach to predict an effective pore radius, since the wetting conditions for *Porefil^®^* are well defined.

To verify the developed correlation between the geometric structure properties and the resulting effective pore radius, the predicted wicking behavior is examined by comparing the experimentally measured curves with the results of the analytical wicking model. Furthermore, to emphasize the accuracy of the correlation, common approaches to determine the effective pore radius are estimated as follows and also used to represent the wicking behavior: The first approach [30] describes the effective capillary pressure without additional corrections, using the geometric pore radius $r_{c}$ from Table 1.

In contrast, the second approach [12,19] describes the capillary pressure using the hydraulic radius $r_{h}$, where the corresponding pore radius $r_{e}$ is determined via $r_{e}=2\;\cdot \;r_{h}$. Here, the hydraulic radius is defined with $r_{h}=2\;\cdot \;\varphi /S_{V}$, where φ represents the porosity and $S_{V}$ is the specific surface area. Both properties are determined as described in [27] in the voxel-based representation of the 3D microstructures.

The other relevant effective properties of the membrane structure, such as the mean pore radius, the mean ligament radius, and the permeability, are extracted on the basis of the CT data as described in Section 2.2.2 and presented in Table 1. Furthermore, the correction factors *F* for samples 1 and 2 are shown in Table 2 with the help of Equation (19). The resulting radii for both samples are summarized in Table 2.

**Table 2:** Effective pore radii, depending on the respective approaches [30] describes the effective capillary pressure without an additional correction, using the geometric radius $r_{c}$. [19], and [12] describes the capillary pressure with the effective radius $r_{e}$, using $r_{e}=2\;\cdot \;r_{h}$. The value $r_{\mathrm{eff}}$ is based on the correction factor *F*, presented in Equation (19).

	$r_{c}$ ( μm)	$r_{e}$ ( μm)	$r_{l}/r_{c}$ (-)	F (-)	$r_{\mathrm{eff}}$ ( μm)
Sample 1	2.16	13.85	0.296	9.6	20.7
Sample 2	1.73	9.26	0.416	7.75	13.4

For the considered membrane samples, the comparison between the analytical prediction and the experimental measurements is shown in Figure 8, where the wicking model is taken from Equation (11). The symbols represent different experimental results, while the curves represent the predictions with different pore radii.

For both samples, significant differences are evident, regarding the three different approaches. When using the geometric pore radius $r_{c}$, the capillary pressure is strongly overestimated in all cases, resulting in a wicking time that is significantly lower than the experimental value. The prediction using the equivalent pore radius $r_{e}$, based on the hydraulic pore radius $r_{h}$, also overestimates the wicking behavior for both samples. In contrast, by using the corrected effective pore radius $r_{\mathrm{eff}}$ from pore-scale simulations and the correction function in Equation (19), an excellent agreement between the experiments and the modeled prediction can be observed.

With a deviation of 7 for sample 1 and 4.8 for sample 2, the wicking time at a wicking length of 4 cm is matched, and the wicking course is well approximated. Furthermore, the coefficient of determination $R^{2}$ was calculated for the statistical validation of all experiments and the respective analytical function. For sample 1 and sample 2, $R^{2}\geq 0.9$ was fulfilled in all cases. This indicates that the wicking course is well approximated, without performing any fit adjustments.

## 4. Conclusions

In this work, a correlation for the determination of effective capillary pore radii in open-pored porous microstructures was derived. For this purpose, the wetting behavior was first simulated in simplified representative structures, using a two-phase phase-field approach. Thus, depending on the structural properties, the different resulting mean surface curvatures of the menisci were evaluated. Based on this simulation study, on the pore-scale, a function for a correction factor *F* was derived, which adjusts the deviation in the effective pore radii between wetting in cylindrical capillaries and porous microstructures. To validate the correlation, the macroscopic wicking behavior in two real PPM samples was calculated and compared to experiments, while the correction factor *F* was calculated based on the mean ligament and the mean pore radius in the respective CT scan.

We demonstrated that the effective radii for the observed samples were about ∼8 times (sample 2) and ∼10 times (sample 1) larger than the geometric mean radius, which was calculated based on the pore size distributions (e.g., see Figure 3e). From a physical point of view, the deviation between the geometrical mean pore radius and the presented effective pore radius originates from the mean surface curvature of the free surface. It is smaller in open-pored porous microstructures than in ideal cylindrical capillaries where the geometrical pore radius and the capillary radius are assumed to be the same. As a result, in porous microstructures, the capillary pressure is smaller, and hence the wicking is slower. In other words, it is evident that this adjustment of the geometrical pore radius is necessary to accurately predict the wicking behavior. With the derived correlation for effective pore radii, no experimental fitting procedures are required to establish further structure–property linkages.

For future work, not only digital twins of a given porous membrane structure but time-dependent physical simulations of the underlying phase separation [52] could be used as well. This would provide a time series of 3D microstructures as database, which brings in combination with the presented computer-aided membrane characterization a tool for direct linking between process parameters for the material production and the resulting wicking behavior. Moreover, by entering the data-driven research in membrane science using research data infrastructures, such as Kadi4Mat [53], far-reaching possibilities for further structure–property linkages and ultimately for the digital design of porous membranes are opened up.

## Figures and Tables

**Table 1:** Effective properties of the two porous nitrocellulose membranes (porosity φ, permeability K, structure parameter $r_{c}$ and $r_{l}$, and the properties of the wicking liquid *Porefil^®^* (surface tension $\gamma_{\mathrm{lg}}$, contact angle $\theta_{e}$, dynamic viscosity η)). The geometric properties are extracted from the reconstructed membrane structures (see Figure 2) by applying the methods described in Section 2.2.2.

		Effective Properties			*Porefil*^®^ Properties [48]		
Sample	φ (-)	K (10${}^{-13}$ ${}^{2}$)	$r_{c}$ ( μm)	$r_{l}$ ( μm)	$\gamma_{\mathrm{lg}}$ (mN m^−1^)	$\theta_{e}$ (${}^{\circ }$)	η (mPa s)
1	0.89	16.83	2.16	0.64	>16.0	>0.0	>2.2
2	0.82	7.78	1.73	0.72			

## Data Availability

Not applicable.
